# Cognitive Function, Mental Health, and Quality of Life in Siblings of Preterm Born Children: Protocol for a Systematic Review

**DOI:** 10.2196/34987

**Published:** 2022-04-14

**Authors:** Wnurinham Silva, Eeva Virtanen, Eero Kajantie, Sylvain Sebert

**Affiliations:** 1 Center for Life Course Health Research Faculty of Medicine University of Oulu Oulu Finland; 2 Department of Population Health Finnish Institute for Health and Welfare (THL) Helsinki Finland; 3 Department of Public Health University of Helsinki Helsinki Finland; 4 PEDEGO Research Unit, Medical Research Center Oulu University Hospital University of Oulu Oulu Finland; 5 Department of Clinical and Molecular Medicine Norwegian University of Science and Tehnology Trondheim Norway; 6 Pediatric Research Centre, Children's Hospital Helsinki University Hospital and University of Helsinki Helsinki Finland

**Keywords:** preterm birth, birth weight, siblings, cognitive, mental health, quality of life, family

## Abstract

**Background:**

Children and adults born preterm are at increased risk of cognitive impairments, mental health disorders, and poorer quality of life. Epidemiological studies have shown that the impact of preterm birth extends to the immediate family members; however, existing research have focused on parents, and little attention has been given to siblings.

**Objective:**

The aim of the systematic review described in this protocol is to synthesize currently available evidence on the impact of exposure to preterm birth (ie, having a sibling born preterm) on cognition, mental health, and quality of life of term born siblings (index child) of preterm born children, and to critically appraise the evidence.

**Methods:**

This protocol outlines a systematic review designed in accordance with the PRISMA-P (Preferred Reporting Items for Systematic Reviews and Meta-Analysis Protocols) checklist. We will include all studies that assess outcomes in siblings of children born preterm. Quantitative and qualitative studies will be eligible for the systematic review, and only studies in English will be included. Firstly, search will be conducted electronically on PubMed, Scopus, Embase, Mednar, and opengrey.eu databases and, secondly, manually in Google Scholar and reference lists. The search strategy will include keywords and synonyms, Boolean operators, and text words (ie, within title and abstract). The team of reviewers will screen the search results, extract data from eligible studies, and critically appraise the studies. Analysis will involve both descriptive and quantitative approaches. Meta-analysis will be conducted if appropriate.

**Results:**

This systematic review was registered on PROSPERO (International Prospective Register of Systematic Reviews) on December 18, 2020, and it is currently in progress. The findings will be synthesized to determine the effect of preterm birth on full-term siblings and the quality of the available evidence.

**Conclusions:**

The evidence derived from this study will shed light on gaps and limitations in the field of preterm birth, more specifically, the effect of preterm birth on full-term siblings. In addition, we hope that understanding the impact of preterm birth on family members will inform targeted interventions and policies for those identified at high risk and how to mitigate health risks.

**Trial Registration:**

PROSPERO International Prospective Register of Systematic Reviews CRD42021222887; https://www.crd.york.ac.uk/prospero/display_record.php?ID=CRD42021222887

**International Registered Report Identifier (IRRID):**

DERR1-10.2196/34987

## Introduction

Global estimates show that 1 in 10 babies are born preterm, and approximately 1 million children die due to complications related to preterm birth every year [1]. Although low- and middle-income countries account for more than half of the world’s preterm births, several high-income countries have experienced an increase of preterm birth rate in the last decades [2].

Preterm birth is defined as infants born alive before 37 completed weeks of gestation or fewer than 259 days from the first date of a woman’s last menstrual period [1,3]. The World Health Organization classifies preterm birth in three subcategories according to gestational age: babies born before 28 completed weeks of gestation are classified as extremely preterm; those born from 28 to 32 weeks are very preterm; and moderate-to-late preterm refer to those born between 32 and 37 weeks of gestation [1]. Prematurity has been associated to risks in neuropsychological functions, such as lower language skills [4], learning disabilities and academic challenges [5], and poor psychiatric and social well-being [6-8]. Research looking at children born moderate and late preterm suggests that when compared with their term-born peers, moderate and late preterm children have poorer social-emotional competence and higher developmental delays and have 3 times higher odds of language impairment [9]. Similarly, extremely preterm children are found to have significantly lower academic attainment [10], lower health-related quality of life [11], and greater risk of having behavioral problems compared with their peers [12].

It has been well reported in the literature, however, that the impact of preterm birth reaches beyond the adverse health outcomes of the preterm child, also affecting the immediate family members. Epidemiological studies have suggested that the poor health outcomes of the family members result from strains posed by preterm birth [13-15]. The adverse effects may differ in time points, according to gender and role in the family, severity of prematurity, the socioeconomic status of the family, and the support made available according to social structures. However, the focus has been placed on the impact of preterm birth on parents and associated environmental stressors [16-21], and little attention has been given to the full-term born sibling of the preterm born child.

Siblings, the first peer group experience, are a bridge to building experiences, which are believed to be pillars for identity development, and traits that will be fundamental to creating relationships and life course experiences outside the family nucleus [22]. In families experiencing crisis or periods of stress, the sibling role is redefined [23]. While there may be opportunities for growth and positive experiences in such environment, there is also a possibility that family dynamics will increase the full-term child’s responsibilities, inhibit their sense of self, and surface health challenges [23]. The association between family dynamics—such as parental stress and depression, interaction, attention, and nurturing—has been recognized to play a crucial role in determining basic cognitive capacities [24], mental health [25], and quality of life [26] in early years and to contribute to adverse health outcomes during childhood and adulthood [26].

Our hypothesis is that siblings of preterm born infants are at risk of poor cognitive, mental health, and quality of life outcomes; that the adverse effects will be more prominent in early life (ie, primary school years); and that its intensity will attenuate into adulthood. A study looking at families with disabled children have reported that internalizing behavior problems are most prevalent among siblings of children with disabilities, compared to control [24]. The study further reported that siblings in families with lower socioeconomic status were more exposed to stressful environments, and consequently at higher risk of adjustment problems [24]. However, little is known about the impact of preterm birth on term born siblings. The existing research investigating the association between preterm birth and the health of siblings has approached this issue mainly in two ways: qualitatively [27-31] and within a familial analysis with focus on parents [28,30-33]. Two qualitative longitudinal studies conducted in Brazil used the perspective of the parents to investigate the impact of preterm birth on term born siblings, during the period of stay at the Neonatal Intensive Care Unit (NICU) [28,34] and up to 36 months of life of the preterm child [34]. Both studies reported an impact on mothers and index children’s relationship as a consequence of the imposed separation resulting from the mother’s need to stay with the preterm newborn at the hospital and placing the siblings under the care of their social support network. In addition, the studies reported that the changes in family dynamics often led to feelings of jealousy, uncertainty, and anxiety conveyed through questions around the preterm born’s health. Another study used direct observation and play to sketch behavioral profiles of 10 children aged between 20 months to 6 years with siblings in the NICU [27]. The author of the study described the term born siblings’ phantasies, defenses, and anxieties during the stay of the preterm sibling in the NICU as accompanied by ambiguous feelings and actions [27]. In their longitudinal study, Saigal et al [33] briefly discussed the impact of preterm birth on siblings, attributing the observed negative impact to reduced parental attention. Similar conclusions were drawn in a phenomenological study by Gaal et al [29], which investigated the experience of 28 term born adults, aged between 17 and 35 years, who grew up with a preterm born sibling. The conclusions were supported by theories arguing that differences in parent-child relationships within families can originate from parents’ different treatment, but also can arise from differences in children’s attributes [35]. In addition to the differentiation treatment, the full-term born siblings also reported feelings of loyalty and responsibility toward their preterm born siblings, despite acknowledgment that preterm siblings disrupted their lives [29].

In synthesis, although the above studies constitute an invaluable contribution to the subject, they suffer from two main limitations. First, they focus on parental reports of sibling health, which provides a potentially subjective and colored depiction of the siblings’ experience. Second, their focus on experiences delimits the conclusions on power and effect of exposure. For example, although the current research suggests that the sibling effect may be important, it is not clear how important the effect may be. It is crucial to understand the degrees to which preterm birth and the different levels of impairment and disability of the preterm born child act as risk factors to the full-term sibling.

Therefore, the purpose of the systematic review will be to synthesize the available evidence investigating and reporting cognition, mental health, and quality of life outcomes of the index child exposed to preterm birth, from early years to adulthood. In addition, we are interested in investigating the effect of outcomes in different ages, and we will critically appraise the evidence. Understanding the impact of preterm birth on term born children may pave the way to targeted, participatory interventions and policies for those identified at high risk and allow for health risks to be mitigated.

## Methods

### Protocol Registration

The protocol is based on the PRISMA-P (Preferred Reporting Items for Systematic Reviews and Meta-Analysis Protocols) checklist. The review protocol has been registered in PROSPERO (International Prospective Register of Systematic Reviews) database (registration CRD42021222887). The review team are 2 PhD candidates (WS and EV), 1 clinician (EK), and 1 epidemiologist (SS). The team offers a multidisciplinary background in maternal and child health, pediatrics, and life-course epidemiology.

### Eligibility Criteria

The traditional systematic review approach based on the Population, Intervention, Comparator, and Outcome framework was used in this protocol to outline the eligibility criteria.

We will include studies in which study the population comprises the index children and their preterm born siblings. Studies where preterm population information on gestational age and birth weight is provided will be included; however, those studies providing only birth weight will only be included when the reported birth weight is lower than 1500 gr. Studies whose population is solely preterm children with major neurodevelopmental impairment (ie, cerebral palsy and mental retardation) will be included, and sensitivity analysis will be conducted where possible. In addition, studies investigating or reporting cognitive outcomes, mental health outcomes, and quality of life outcomes of the index children will be included. Studies including survivor and nonsurvivor preterm siblings will also be included. We are interested in research studies using school records, health professional and parental reports, and reports by the index children. Finally, only studies in English will be included, for reproducibility and to allow both reviewers to contribute equally to the review process.

Studies reporting only the preterm sibling outcomes with term-born siblings as controls will be excluded, and studies defining preterm birth as birth weight of lower than 2500 gr, but without indication of gestational age, will also be excluded.

### Outcomes

The outcomes of the review will be grouped under the following headings: (1) cognitive outcomes, which include cognitive test scores, rates of cognitive impairment, reported learning difficulties, and related traits; (2) mental health outcomes, which include psychiatric diagnoses, subthreshold symptoms, and behavioral traits predicting mental health disorders; and (3) quality of life outcomes, which include standardized quality of life questionnaires on life events such as education, occupation, or starting a family.

### Information Sources

Studies will be identified using two approaches. First, electronic searches will be conducted on PubMed, Scopus, and Embase electronic databases. For grey literature, we will be using opengrey.eu and Mednar databases. Second, we will conduct manual searches on Google Scholar and on reference lists of the relevant articles obtained on electronic databases, to retrieve additional relevant articles. The search will be conducted by 2 reviewers, WS and EV, in consultation with an expert librarian.

### Search Strategies

The search strategy includes keywords (ie, MeSH [Medical Subject Headings] terms), a set of synonyms for the keywords, and text words (ie, within title and abstract) which will be combined, as illustrated in Multimedia Appendices 1 and 2. These search terms will be entered into the databases and will be truncated as appropriate recurring to use of Boolean operators.

### Study Selection

Literature search results will be transferred to a reference management software and screened for duplicates. The review of the studies will be conducted by WS and EV in two stages using the review’s eligibility criteria. The first phase of the selection process consists of the screening of titles and abstracts. The second phase will involve examination of the full text for compliance of studies with eligibility criteria. In case of disagreement at any of the above stages, a third reviewer will be brought in for a decision. In case of insufficient information in the articles, WS will contact the authors for clarification. There will be given 2 weeks to receive a reply before the article is excluded for lack of reply and insufficient information. The data will be recorded in a Microsoft Excel spreadsheet that is shared between the reviewers and supervisory team. Records of all searches in the different databases will be kept, and the search process will be presented in a PRISMA flow chart to show the details of the selection process.

### Data Extraction

Two reviewers will conduct data extraction in order to minimize bias and reduce error. For all records, the following details will be recorded: title, authors, publication date, study design, country, population characteristics (including number of participants, birth subcategories, and weight), methods, outcome data, and time point of measurements (Table 1). Data will be organized in a way that will support and enable interpretation of results in the data synthesis stage. A standardized data extraction form will be created for use among reviewers. The data extraction form will be piloted by EK and SS.

**Table 1:** Data extraction variables.

Variables	Content
Study	Authors Title Publication status Publication date Study aims Study design Country
Classifications	Prematurity subcategory model used Prematurity subcategories description Birth weight measure used
Age	Age of preterm children at time of study
Population characteristics	Population type Population size
Time point	Time point of measurement
Outcomes	Outcomes reported
Results	Significance of evidence (statistical)

### Critical Appraisal

All selected studies will be fully evaluated for quality. Critical appraisal will be conducted by WS and EV. A quality assessment tool based on the Critical Appraisal Skills Programme and the Joanna Briggs Institute Critical Appraisal tool will be used to assess the quality and conduct the validity of the selected studies. The tool has been tailored to allow evaluation of studies using different methodologies. The studies will be assessed for internal and external validity and bias.

### Data Synthesis

Data synthesis will be conducted using both quantitative and qualitative methods. A summary table of all included studies will be provided, and interpretation of findings will include a summary of inclusion and exclusion criteria. This will be accompanied by a flow diagram including the number of unique records identified by the searches, records excluded after preliminary screening, records retrieved in full text, studies excluded after assessment of the full text, and studies meeting the eligibility criteria for the review. Methods and design of the studies will also be analyzed, and the potential study bias discussed. Evidence will be graded using the GRADE (grading of recommendations, assessment, development, and evaluation) system, and a summary of findings table presented.

### Quantitative Data Synthesis

If 3 or more studies are available for analysis, meta-analysis will be conducted to generate a more precise estimate of the magnitude of effect on health outcomes of parents and full-term siblings, using R statistical software (The R Foundation). The most appropriate statistical analysis will be chosen and used, considering type of studies and heterogeneity of the final studies. If appropriate, subgroup analysis will be carried out to explore differences between preterm subcategories, provided the data presented on literature allow such examination.

### Qualitative Data Synthesis

Qualitative evidence will be analyzed using the metastudy methodology [36]. This approach is conducted in two stages, which are the following: (1) analysis stage, which involves analyzing theory (metatheory), methods (metamethod), and findings (metadata analysis) of the primary studies; and (2) meta-synthesis stage, which involves an in-depth interpretation of the results to create an understanding of the topic being studied [36,37].

The metamethod and metatheory analysis will be guided by our aim to critically appraise the available evidence on cognition, mental health, and quality of life outcomes of the index child exposed to preterm birth. In addition, we will evaluate the theoretical framework used in the qualitative study, how these theories support the methods used, and the conclusions achieved in each study. As part of the metadata analysis, we will critically interpret the results and find similarities and discrepancies between the included studies [36].

### Amendments

Any protocol amendments will be properly documented by the authors, including date of changes, description of the changes, and rationale for changes.

### Ethics and Dissemination

Ethical approval is not needed for this systematic review because primary data will not be collected. The systematic review will be published in a peer-reviewed journal.

### Patient and Public Involvement in Research

It was not appropriate or possible to involve patients or the public in the design, conduct, reporting, or dissemination plans of this protocol and respective systematic review.

## Results

This systematic review was registered on PROSPERO on December 18, 2020, and it is currently in progress. The findings will be synthesized to determine the effect of preterm birth on full-term siblings and the quality of the available evidence.

## Discussion

Our systematic review aims to synthesize the best available evidence on the long-term trajectory of the impact of preterm birth on health outcomes of index children and offer a detailed appraisal of the methodological quality of the research. This review is the first attempt to synthesize and critically appraise evidence on the impact of exposure to preterm birth on full-term born siblings, to the best of our knowledge. In doing so, we reinforce the importance of recognizing extended familial factors when considering preterm birth. This systematic review will follow PRISMA guidelines, and we aim to follow high-standard quality; however, we recognize the ever-existing risk of bias and that this may represent a study limitation. We will implement several strategies to minimize risk of bias, such as blinded review, as well as discussions between reviewers and within the review team at each stage of the review and validate data to ensure reliability. It is expected that a critical evaluation of the existing literature will provide an insight into the gaps and limitations of the longitudinal study of preterm birth and inform future works in this field.

## Appendix

Multimedia Appendix 1 Pubmed search.

Multimedia Appendix 2 Scopus search.

## Abbreviations

GRADE: grading of recommendations, assessment, development, and evaluation
MeSH: Medical Subject Headings
NICU: Neonatal Intensive Care Unit
PRISMA-P: Preferred Reporting Items for Systematic Reviews and Meta-Analysis Protocols
PROSPERO: International Prospective Register of Systematic Reviews

## Data Availability

All data relevant to the study are included in the article or uploaded as supplementary information.

## References

[1] Preterm Birth. World Health Organization.

[2] Vogel JP, Chawanpaiboon S, Moller A, Watananirun K, Bonet M, Lumbiganon P (2018). The global epidemiology of preterm birth. Best Pract Res Clin Obstet Gynaecol.

[3] World Health Organization, March of Dimes, Save the Children (2012). Born too soon: the global action report on preterm birth.

[4] Kunnari S, Yliherva A, Paavola L, Peltoniemi OM (2012). Expressive language skills in Finnish two-year-old extremely- and very-low-birth-weight preterm children. Folia Phoniatr Logop.

[5] Lind A, Nyman A, Lehtonen L, Haataja L (2020). Predictive value of psychological assessment at five years of age in the long-term follow-up of very preterm children. Child Neuropsychol.

[6] Bilgin A, Mendonca M, Wolke D (2018). Preterm Birth/Low Birth Weight and Markers Reflective of Wealth in Adulthood: A Meta-analysis. Pediatrics.

[7] Kajantie E, Strang-Karlsson S, Evensen KAI, Haaramo P (2019). Adult outcomes of being born late preterm or early term - What do we know?. Semin Fetal Neonatal Med.

[8] Vääräsmäki M, Sipola-Leppänen M, Tikanmäki M, Matinolli H, Pesonen A, Räikkönen K, Järvelin M, Hovi P, Kajantie E (2015). Independent Living and Romantic Relations Among Young Adults Born Preterm. PEDIATRICS.

[9] Cheong JL, Doyle LW, Burnett AC, Lee KJ, Walsh JM, Potter CR, Treyvaud K, Thompson DK, Olsen JE, Anderson PJ, Spittle AJ (2017). Association Between Moderate and Late Preterm Birth and Neurodevelopment and Social-Emotional Development at Age 2 Years. JAMA Pediatr.

[10] Johnson S, Wolke D, Hennessy E, Marlow N (2011). Educational outcomes in extremely preterm children: neuropsychological correlates and predictors of attainment. Dev Neuropsychol.

[11] Saigal S, Ferro MA, Van Lieshout RJ, Schmidt LA, Morrison KM, Boyle MH (2016). Health-Related Quality of Life Trajectories of Extremely Low Birth Weight Survivors into Adulthood. J Pediatr.

[12] Linsell L, Johnson S, Wolke D, Morris J, Kurinczuk JJ, Marlow N (2019). Trajectories of behavior, attention, social and emotional problems from childhood to early adulthood following extremely preterm birth: a prospective cohort study. Eur Child Adolesc Psychiatry.

[13] Henderson J, Carson C, Redshaw M (2016). Impact of preterm birth on maternal well-being and women's perceptions of their baby: a population-based survey. BMJ Open.

[14] Lakshmanan A, Agni M, Lieu T, Fleegler E, Kipke M, Friedlich PS, McCormick MC, Belfort MB (2017). Health Qual Life Outcomes.

[15] Saigal S, Pinelli J, Streiner DL, Boyle M, Stoskopf B (2010). Impact of extreme prematurity on family functioning and maternal health 20 years later. Pediatrics.

[16] Ionio C, Colombo C, Brazzoduro V, Mascheroni E, Confalonieri E, Castoldi F, Lista G (2016). Mothers and Fathers in NICU: The Impact of Preterm Birth on Parental Distress. Eur J Psychol.

[17] Latva R, Korja R, Salmelin RK, Lehtonen L, Tamminen T (2008). How is maternal recollection of the birth experience related to the behavioral and emotional outcome of preterm infants?. Early Hum Dev.

[18] Lundqvist P, Weis J, Sivberg B (2019). Parents' journey caring for a preterm infant until discharge from hospital-based neonatal home care-A challenging process to cope with. J Clin Nurs.

[19] O'Donovan A, Nixon E (2019). "Weathering the storm:" Mothers' and fathers' experiences of parenting a preterm infant. Infant Ment Health J.

[20] Russell G, Sawyer A, Rabe H, Abbott J, Gyte G, Duley L, Ayers S, “Very Preterm Birth Qualitative Collaborative Group” (2014). Parents' views on care of their very premature babies in neonatal intensive care units: a qualitative study. BMC Pediatr.

[21] Treyvaud K, Lee KJ, Doyle LW, Anderson PJ (2014). Very preterm birth influences parental mental health and family outcomes seven years after birth. J Pediatr.

[22] Caplan R (2011). Someone Else Can Use This Time More Than Me: Working with College Students with Impaired Siblings. Journal of College Student Psychotherapy.

[23] Lamorey S (1999). Parentification of Siblings of Children With Disability or Chronic Disease. Burdened children: Theory, research, and treatment of parentification.

[24] Nampijja M, Kizindo R, Apule B, Lule S, Muhangi L, Titman A, Elliott A, Alcock K, Lewis C (2018). The role of the home environment in neurocognitive development of children living in extreme poverty and with frequent illnesses: a cross-sectional study. Wellcome Open Res.

[25] Knudsen EI, Heckman JJ, Cameron JL, Shonkoff JP (2006). Economic, neurobiological, and behavioral perspectives on building America's future workforce. Proc Natl Acad Sci U S A.

[26] Shonkoff JP, Garner AS, Committee on Psychosocial Aspects of ChildFamily Health, Committee on Early Childhood‚ Adoption‚Dependent Care, Section on Developmental Behavioral Pediatrics (2012). The lifelong effects of early childhood adversity and toxic stress. Pediatrics.

[27] Camhi C (2005). Siblings of premature babies: Thinking about their experience. Infant Observation.

[28] Carvalho SC, Facio BC, Souza BFD, Abreu-D'Agostini FCP, Leite AM, Wernet M (2019). Maternal care in the preterm child's family context: A comprehensive look towards the sibling. Rev Bras Enferm.

[29] Gaal BJ, Pinelli J, Crooks D, Saigal S, Streiner DL, Boyle M (2010). Outside looking in: the lived experience of adults with prematurely born siblings. Qual Health Res.

[30] Stjernqvist KM (1992). Extremely low birth weight infants less than 901 g. Impact on the family during the first year. Scand J Soc Med.

[31] Taylor LS (2008). A Rites of Passage analysis of the families' experience of premature birth. Journal of Neonatal Nursing.

[32] Johnston SR (2010). Sibling adjustment following the birth of a premature infant. ETD Collection for Pace University.

[33] Saigal S, Burrows E, Stoskopf BL, Rosenbaum PL, Streiner D (2000). Impact of extreme prematurity on families of adolescent children. J Pediatr.

[34] Mousquer PN, Leão LCDS, Kepler DF, Piccinini CA, Lopes RDCS (2014). Mãe, cadê o bebê? Repercussões do nascimento prematuro de um irmão. Estud. psicol. (Campinas).

[35] Deater-Deckard K, Petrill SA (2004). Parent-child dyadic mutuality and child behavior problems: an investigation of gene-environment processes. J Child Psychol Psychiatry.

[36] Paterson B (2001). Meta-study of Qualitative Health Research: A Practical Guide to Meta-analysis and Meta-synthesis. Emerald Insight.

[37] Zhao S (2016). Metatheory, Metamethod, Meta-Data-Analysis: What, Why, and How?. Sociological Perspectives.

