# Supplementary material for: Cognitive Function, Mental Health, and Quality of Life in Siblings of Preterm Born Children: Protocol for a Systematic Review
Source: JMIR Res Protoc. 2022 Apr 14;11(4):e34987. doi: 10.2196/34987 (PMC9052026; doi:10.2196/34987)
Supplement: Multimedia Appendix 1 [file resprot_v11i4e34987_app1.pdf]

Multimedia Appendix 1. Pubmed search.

("Siblings"[MeSH Terms] OR "brother*"[Text Word] OR "sister*"[Text Word] OR "sibling*"[Text Word]) **AND** ("infant, extremely premature"[MeSH Terms] OR "Premature Birth"[MeSH Terms] OR "infant, premature"[MeSH Terms] OR ("infant, extremely low birth weight"[MeSH Terms] OR "infant, very low birth weight"[MeSH Terms] OR "Gestational Age"[MeSH Terms]) OR "infant, low birth weight"[MeSH Terms] OR "preterm"[Text Word] OR "Gestational Age"[Text Word] OR "birth weight"[Text Word] OR "premat*"[Text Word]) **AND** ("Social Support"[MeSH Terms] OR "support"[Text Word] OR "burden"[Text Word] OR "routine"[Text Word] OR ("stress disorders, traumatic"[MeSH Terms] OR "stress, psychological"[MeSH Terms] OR "Anxiety"[MeSH Terms] OR ("Depression"[MeSH Terms] OR "Depressive Disorder"[MeSH Terms]) OR "Aggression"[MeSH Terms] OR ("Attention Deficit and Disruptive Behavior Disorders"[MeSH Terms] OR "disruptive, impulse control, and conduct disorders"[MeSH Terms] OR "Problem Behavior"[MeSH Terms]) OR "Impulsive Behavior"[MeSH Terms] OR ("Emotions"[MeSH Terms] OR "Guilt"[MeSH Terms]) OR ("Affect"[MeSH Terms] OR "Mood Disorders"[MeSH Terms]) OR ("Conduct Disorder"[MeSH Terms] OR "Behavior"[MeSH Terms] OR "Criminal Behavior"[MeSH Terms]) OR "Personality"[MeSH Terms] OR "stress"[Text Word] OR "Anxiety"[Text Word] OR "depressi*"[Text Word] OR "aggressi*"[Text Word] OR "disrupt*"[Text Word] OR "impulsiv*"[Text Word] OR "feel*"[Text Word] OR "mood"[Text Word] OR "conduct"[Text Word] OR "Personality"[Text Word] OR "need*"[Text Word] OR "self*"[Text Word] OR "behav*"[Text Word] OR "care"[Text Word]) OR ("Neurodevelopmental Disorders"[MeSH Terms] OR ("Intelligence"[MeSH Terms] OR "Intellectual Disability"[MeSH Terms] OR "Interpersonal Relations"[MeSH Terms]) OR ("Education"[MeSH Terms] OR "Educational Status"[MeSH Terms]) OR ("Academic Failure"[MeSH Terms] OR "Academic Success"[MeSH Terms] OR "Academic Performance"[MeSH Terms] OR "Learning Disabilities"[MeSH Terms]) OR "Schools"[MeSH Terms] OR ("Developmental Disabilities"[MeSH Terms] OR "Language Development Disorders"[MeSH Terms] OR "child development disorders, pervasive"[MeSH Terms]) OR "neurodevelop*"[Text Word] OR "cognit*"[Text Word] OR "intelligen*"[Text Word] OR "intellect*"[Text Word] OR "education*"[Text Word] OR "academic"[Text Word] OR "school"[Text Word] OR "disorder"[Text Word] OR "develop*"[Text Word] OR "disab*"[Text Word]) OR ("Quality of Life"[MeSH Terms] OR "stress, psychological"[MeSH Terms] OR ("Psychosocial Deprivation"[MeSH Terms] OR "Intellectual Disability"[MeSH Terms]) OR "Mental Health"[MeSH Terms] OR "Quality of Life"[Text Word] OR "psychological"[Text Word] OR "Mental Health"[Text Word]))
