# Supplementary material for: Cognitive Function, Mental Health, and Quality of Life in Siblings of Preterm Born Children: Protocol for a Systematic Review
Source: JMIR Res Protoc. 2022 Apr 14;11(4):e34987. doi: 10.2196/34987 (PMC9052026; doi:10.2196/34987)
Supplement: Multimedia Appendix 2 [file resprot_v11i4e34987_app2.pdf]

Multimedia Appendix 2. Scopus search.

TITLE-ABS-KEY (sibling* OR brother* OR sister*)
AND
TITLE-ABS-KEY (preterm OR "birth weight" OR premat* OR "gestational age")
AND
TITLE-ABS-KEY ("Quality of Life" OR psychological OR psychos* OR "mental health")
OR
TITLE-ABS-KEY
(feel* OR stress OR anxiety OR depressi* OR aggress* OR mood OR disrupt* OR impulsiv* OR conduct OR personality OR behav* OR need* OR self*)
OR
TITLE-ABS-KEY
(education OR academic OR school OR cognit* OR neurodevelop* OR intellect* OR intelligen* OR disab*)
OR
TITLE-ABS-KEY (support* OR burden OR routine OR care)
